# Quantitative analysis of numerical solvers for oscillatory biomolecular system models

**DOI:** 10.1186/1471-2105-9-S6-S17

**Published:** 2008-05-28

**Authors:** Chang F Quo, May D Wang

**Affiliations:** 1 Wallace H. Coulter Department of Biomedical Engineering, Georgia Institute of Technology and Emory University, Atlanta, GA 30332, USA; 2 School of Electrical and Computer Engineering, Georgia Institute of Technology, Atlanta, GA 30332, USA; 3 Hematology and Oncology, Winship Cancer Institute, Emory University, Altanta, GA 30322, USA; 4 The Parker H. Petit Institute for Bioengineering and Bioscience, Georgia Institute of Technology, Atlanta, GA 30332, USA

## Abstract

**Background:**

This article provides guidelines for selecting optimal numerical solvers for biomolecular system models. Because various parameters of the same system could have drastically different ranges from 10^-15^ to 10^10^, the ODEs can be stiff and ill-conditioned, resulting in non-unique, non-existing, or non-reproducible modeling solutions. Previous studies have not examined in depth how to best select numerical solvers for biomolecular system models, which makes it difficult to experimentally validate the modeling results. To address this problem, we have chosen one of the well-known stiff initial value problems with limit cycle behavior as a test-bed system model.  Solving this model, we have illustrated that different answers may result from different numerical solvers. We use MATLAB numerical solvers because they are optimized and widely used by the modeling community. We have also conducted a systematic study of numerical solver performances by using qualitative and quantitative measures such as convergence, accuracy, and computational cost (i.e. in terms of function evaluation, partial derivative, LU decomposition, and "take-off" points). The results show that the modeling solutions can be drastically different using different numerical solvers. Thus, it is important to intelligently select numerical solvers when solving biomolecular system models.

**Results:**

The classic Belousov-Zhabotinskii (BZ) reaction is described by the Oregonator model and is used as a case study. We report two guidelines in selecting optimal numerical solver(s) for stiff, complex oscillatory systems: (i) for problems with unknown parameters, ode45 is the optimal choice regardless of the relative error tolerance; (ii) for known stiff problems, both ode113 and ode15s are good choices under strict relative tolerance conditions.

**Conclusions:**

For any given biomolecular model, by building a library of numerical solvers with quantitative performance assessment metric, we show that it is possible to improve reliability of the analytical modeling, which in turn can improve the efficiency and effectiveness of experimental validations of these models.  Also, our study can be extended to study a variety of molecular-level system models for human disease diagnosis and therapeutic treatment.

## Background

Complex biochemical systems have been described by many models [1-4], but these modeling studies have not been used to address biomedical problems of practical or clinical interest. One major hurdle is that when describing a non-observable biomolecular system, the set of ordinary differential equations (ODEs) often do not have closed-form analytical solutions. Even when numerical solutions are proposed, there are no standard quantitative metrics to verify the efficacy of a numerical solution in approximating the true solution. Thus, there is an urgent need to study numerical solver behavior in biomolecular systems modeling using both qualitative and quantitative performance measures.

**Figure 1:**
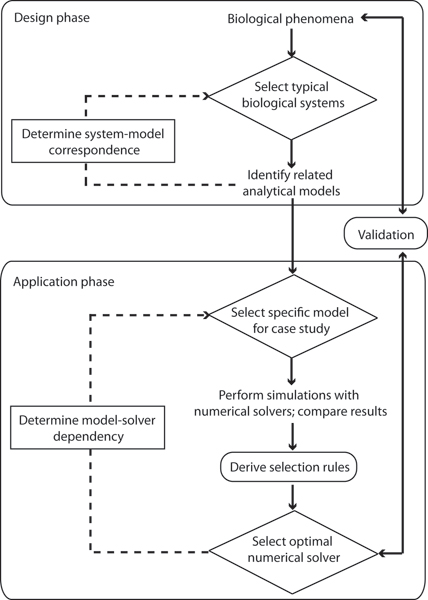
**Workflow diagram for systematic study of numerical solver behavior**. 2 primary phases are identified in this systematic study – design and application. This study focuses on the application phase; specifically, to derive rules to guide the selection of optimal numerical solver(s).

Biomolecular system modeling is an iterative process that depends on robust and accurate numerical solvers for future experimental validation. We aim to determine the optimal numerical solver(s) for a particular system model, by applying various numerical solvers to the model and then comparing the numerical solutions. The workflow for such a systematic study is shown in Figure 1.

• In the application phase, we select the optimal numerical solver(s) (ideally based on a weighted decision from performance measures) to elucidate model-solver dependency (that is, to determine the characteristics of numerical solvers that can express model behavior reasonably well).

• In the design phase, we identify and incorporate significant features of similar biomolecular systems into concrete analytical models. This is to determine a system-model correspondence.

• Based on specific model characteristics, we apply the optimal numerical solver(s) to obtain the solution that best approximates the true system behavior.

• We validate the model through experimentation by comparing empirical results with the simulated model results by using the optimal solver(s).

• We improve the optimal numerical solver(s)  selection to correct modeling errors by reducing discrepancies between experimental results and modeling simulations.

As a test-bed, we have used the Belousov-Zhabotinskii (BZ) reaction system, which is represented by the Oregonator model [4] as the test system. In the following sections, we first describe the system model and numerical solvers implemented; we then present and discuss the results obtained using qualitative and quantitative performance measures; and we conclude by laying out plans for related future work. Finally, we will determine simple rules to help select the optimal numerical solver(s) given stiff problems. These rules can help researchers decide if there exists an optimal numerical solver(s) for a given system or if there are only sub-optimal solvers.

## Results

Two sets of simulations were performed under different conditions of error tolerance – (a) relaxed relative error tolerance (RET) i.e. in MATLAB, 'RelTol' = 10^-3 ^and (b) strict relative error tolerance (SET) i.e. 'RelTol' = 10^-6 ^; other invariant conditions are a parameter set {s = 100, w = 3.835, q = 10^-6^, f = 1.1} and initial conditions {*α *= 20, *η *= 1.1, *ρ *= 20}. Simulations were run for 200 time units.

**Figure 2:**
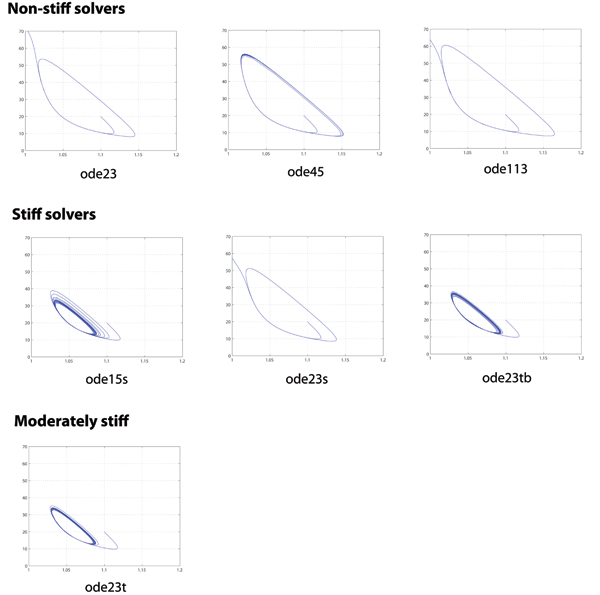
**Phase plots of *ρ*(Z) vs. *η*(Y) under RET ('RelTol' = 10^-3^)**. The plots are physically dimensionless on both axes, but may be considered analogous to units of concentration. x-axis: [1,1.2]; y-axis: [0,70]. Limit cycle behavior was observed for all numerical solvers under SET ('RelTol' = 10^-6^).

**Figure 3:**
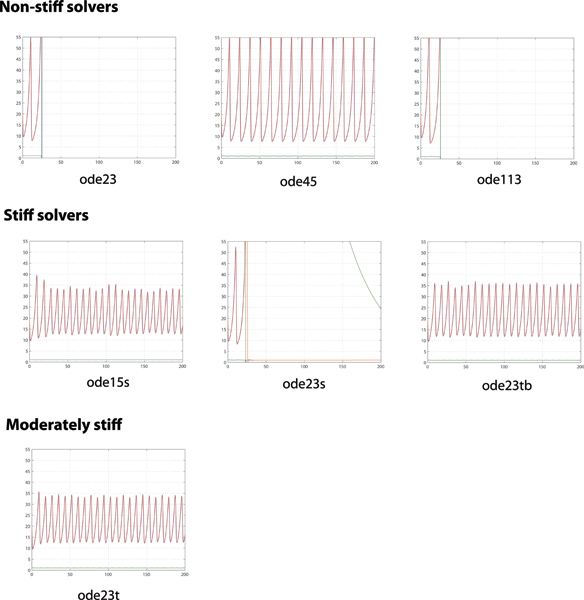
**Time-series solutions of *α*(X), *η*(Y) and *ρ*(Z) under RET ('RelTol' = 10^-3^)**. x-axis: [0,200]; y-axis: [0,55]. *α*(X): blue, *η*(Y): green and *ρ*(Z): red. Limit cycle behavior was observed for all numerical solvers under SET ('RelTol' = 10^-6^) (data not shown).

Figures 2 and 3 are visual representations of simulation results under RET. Respectively, Figure 2 shows 2-D phase plots of pairs of reactant species and Figure 3 shows time-series solutions of all three reactant species. These results are based on the assumed ability of the numerical solvers in MATLAB to handle stiff problems as shown in Table 1. In Figure 3, only the phase plots of *ρ*(Z) vs. *η*(Y) are presented for brevity; the trends presented are representative of other combination pairs.

**Table 1:** Overview of MATLAB numerical solvers [5,18]

Solver	Problem Type	Step	Order	Accuracy	Explicit/Implicit
ode23	Non-stiff	Single	2^nd^/3^rd^	Low	Explicit
ode45			4^th^	Medium	
ode113		Multi	1^st^–13^th^	Low to High	
ode23t	Moderately stiff	Single	2^nd^/3^rd^	Low	Implicit
ode15s	Stiff	Multi	1^st^–5^th^	Low to Medium	
ode23s		Single	2^nd^/3^rd^	Low	
ode23tb			2^nd^/3^rd^	Low	

Based on these performance measures, we propose two simple guidelines to select the optimal numerical solver(s) for solving models of stiff, complex oscillatory biochemical systems.

1. For problems with unknown parameters, ode45 is the optimal choice regardless of relative error tolerance.

2. For known stiff problems, both ode113 and ode15s are good choices under strict relative error tolerance.

### Qualitative metrics

From Figure 2 under RET, we observe that the expected limit cycle (LC) behavior only exists in some solvers. For example, among the non-stiff solvers, only ode45; among the stiff solvers, only ode15s and ode23tb; and the moderately stiff solver ode23t. LC behavior is observed from all solvers under SET.

In Figure 3, the time-series solutions are logically parallel to the 2-D phase plots in Figure 2. In addition, the non-stiff solvers ode23, ode113 and stiff solver ode23s produce solutions that quickly depart from the expected limit cycle behavior and become unstable. For reference purposes, we define a 'take-off' point as the pivotal time-point when the numerical solution first departs radically, by visual inspection of time-series data, from expected stable limit cycle behavior. Furthermore, of these solutions in Figure 3, the stiff solvers ode15s and ode23tb produce oscillations with varying amplitudes and periods with pseudo-random sequence while the oscillations from ode45 and ode23t are relatively uniform throughout the period of simulation. In addition, where limit cycle behavior is observed under SET, the amplitudes of oscillations are similar for *α *and *ρ*, ranging between [43.3, 50.7] while the corresponding period ranges between [12.4, 13.6].

From Figures 2 and 3, we observe significant differences in the numerical solutions obtained using different numerical solvers on the same system model under similar operating conditions (e.g. model parameters and initial conditions). These observations are summarized in Tables 2 and 3, together with other qualitative and quantitative performance metrics.

**Table 2:** Quantitative performance measures under RET ('RelTol' = 10^-3^)

Solver Type	Non-stiff			Stiff			Moderately stiff
**Solver**	**ode23**	**ode45**	**ode113**	**ode15s**	**ode23s**	**ode23tb**	**ode23t**

Successful steps	-	785	-	827	-	695	859
Failed attempts	-	30	-	147	-	141	127
Function evaluations	-	4891	-	2222	-	3757	2439
Partial derivatives	n/a			43	-	71	63
LU decompositions				272	-	370	377
Solutions of linear systems				2049	-	4218	2186
Convergence	No	Yes	No	Yes	No	Yes	Yes
Take-off point^♮^	Variable	-	25.55	-	23.05	-	-
Period^♯^	-	13.5	-	8.5	-	8.9	8.5
Amplitude^♯^	-	49.7	-	23.4	-	25.6	21.7

^♮^'Take-off' point defined as the pivotal time-point when the numerical solution first departs radically from expected limit cycle behavior, by visual inspection.

^♯^Period and amplitude data for *α*(X) and *ρ*(Z) from Figure 3.

**Table 3:** Quantitative performance measures under SET ('RelTol' = 10^-6^)

Solver Type	Non-stiff			Stiff			Moderately stiff
**Solver**	**ode23**	**ode45**	**ode113**	**ode15s**	**ode23s**	**ode23tb**	**ode23t**

Successful steps	5821	2306	2434	2421	10996	6609	8459
Failed attempts	0	0	93	240	0	17	5
Function evaluations	17464	13837	4962	5242	65978	24311	15564
Partial derivatives	n/a			30	10996	10	1
LU decompositions				499	10996	458	461
Solutions of linear systems				5121	32988	30887	15559
Convergence	Yes	Yes	Yes	Yes	Yes	Yes	Yes
Period^♯^	13.6	13.6	13.7	13.4	13.6	12.4	12.4
Amplitude^♯^	50.6	50.5	50.7	48.4	50.5	44.0	43.3

^♯^Period and amplitude data for *α*(X) and *ρ*(Z) from Figure 3.

### Quantitative metrics

**Figure 4:**
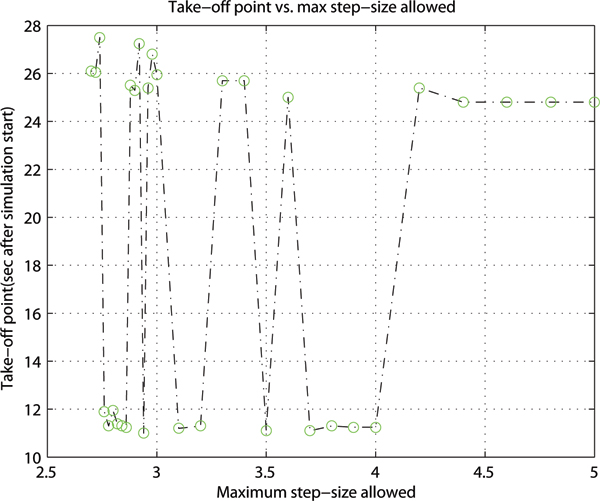
**'Take-off point' variation for ode23 simulation under RET ('RelTol' = 10^-3^)**. The 'take-off' point is defined as the pivotal time-point when the numerical solution first departs radically from expected stable limit cycle behavior. This is determined by visual inspection of the time-series solution. Adaptive step-sizes allowed in implementation may account for the variation of the 'take-off' point with respect to the maximum step-size allowed.

Comparing statistics in Table 2 under RET, ode45 is the only non-stiff solver that produces a convergent solution; ode15s is the least costly stiff solver based on the number of function evaluations. Among all implicit solvers, ode15s is the optimal stiff solver because it had to compute the least number of partial derivatives, LU decompositions and solutions of linear systems. The moderately stiff solver ode23t performs the next best in this group of implicit solvers. Between implicit and explicit solvers, where convergent solutions to LC behavior were determined, the observed period and amplitude of oscillations is significantly different: (13.5, 49.7) from ode45 as opposed to an average of (8.6, 23.6) from ode15s, ode23tb and ode23t. Where numerical solutions do not converge, the 'take-off' point is consistent for ode113 (25.55 time units) and ode23s (23.05), but varies for ode23 depending on the maximum step-size allowed. This phenomenon is further examined in Figure 4.

Comparing statistics in Table 3 under SET, in terms of the number of function evaluations, among explicit solvers ode113 is the least costly i.e. it performed the least number of function evaluations. Among implicit solvers, ode15s is optimal in terms of computational costs i.e. computing the least number of partial derivatives, LU decompositions and solutions of linear systems.

Comparing statistics across Tables 2 and 3, only ode45 provided consistent results in terms of the period and amplitude of oscillation – (13.5, 49.7) under RET and (13.6, 50.5) under SET. Among implicit solvers, ode15s performed the best in terms of computational cost. From Table 2, only one of three explicit solvers and 3 of 4 implicit solvers produced convergent numerical solutions under RET. From Table 3, all numerical solvers produced convergent numerical solutions to LC behavior when the relative error tolerance was stricter under SET.

Following the departure of the numerical solution from expected limit cycle behavior, we observe that the 'take-off' point for non-stiff solver ode23 under RET varies based on the maximum step-size (i.e. 'MaxStep' in MATLAB) allowed. In all other simulations performed for this study, the simulation option 'MaxStep' is set by default with the rule 'MaxStep' = 0.1*abs(t_0 _- t_f_) [5] where t_0 _is the simulation start time, set at 0 units; t_f _is the end time, set at 200 time units. In fact, the period of simulation dictates the maximum step-size allowed. The actual step-sizes taken are more directly controlled by the relative error tolerance 'RelTol'. The relation of the 'take-off' point with respect to the 'MaxStep' allowed for ode23 simulations is shown in Figure 4. From Figure 4, the 'take-off' point varies between ~11 and ~26 time units with 'MaxStep' ≤ 4.2 units. The 'take-off' point remains constant at ~24.6 if the'MaxStep' is >4.2.

## Discussion

Numerical solvers have various theoretical formulations such as single- vs. multi-step, low- vs. high-order, explicit vs. implicit solvers [6-8]. To systematically compare the numerical solvers, we have examined qualitative performance measures such as convergence and accuracy, and quantitative performance measures such as computational cost in terms of function evaluations, partial derivatives, LU decompositions, solutions of linear systems, 'take-off' points, period and amplitude of oscillation.

Adaptive schemes allow savings in computational cost without compromising accuracy, i.e., the solvers take smaller step-sizes when the results change rapidly, or larger step-sizes when the results move slowly. With a specified tolerance interval for the magnitude of step errors, the step-size may be adjusted so that smaller steps are taken where the step error is large and vice versa. This adaptive step-size feature is studied by performing simulations under 2 conditions: (a) relaxed relative error tolerance (RET), i.e. in MATLAB, 'RelTol' = 10^-3^, and (b) strict relative error tolerance (SET), 'RelTol' = 10^-6^.

Single-step numerical solvers depend only on one preceding time-point i.e. only y(t_n-1_) is required to obtain y(t_n_). Hence, trends from additional preceding time-points do not influence the solution of the immediate step. On the other hand, multi-step solvers require multiple preceding time-points to determine y(t_n_). As such, multi-step methods are less sensitive to initial conditions compared to single-step methods in general. In the case of single-step Runge-Kutta (RK) methods – that form the basis for ode23, ode45 and ode23tb – the interval between t_n-1 _and t_n _are further divided into subintervals based on the order of the RK method. This subdivision also helps to control error propagation at successive steps, leading to increased stability in the solution. Thus, with the exception of ode23 where the solution quickly diverges, RK methods in general are a popular choice for numerical solvers.

The explicit solvers compute y(t_n_) using other known values, while the implicit solvers require an iterative process to solve y(t_n_) instead. Convergence is not guaranteed in this iterative process and depends heavily on the termination criteria. On the other hand, because this iterative process refines the solution at each step, implicit solvers are generally more suited to solve stiff problems. An unexpected result occurs with ode23s, where the solution becomes unstable under RET.

The tradeoff for obtaining a high order of accuracy is increased computational cost. For example, the variable-order solver ode113 is capable of accuracy up to the 13th order. However, to achieve this accuracy, it performs extensive function evaluations depending on the highest order of the derivatives required. On the other hand, low-order solvers such as ode23 perform simpler function evaluations that are limited to computing the 3rd-order derivative.

From this comparison, our results suggest ode45 and ode15s are better numerical solvers when dealing with stiff systems of nonlinear ODEs. ode45 is an explicit RK method of order 4 while ode15s is variable-order capable of 1st–5th order accuracy. Furthermore, low- to medium-order methods appear to provide more accurate solutions compared to high-order methods. In addition, results suggest that under relaxed relative error tolerance, implicit solvers may handle stiff problems relatively better than explicit solvers; this is consistent with established guidelines in handling stiff problems. However, when the relative error tolerance is strict, all numerical solutions studied here converge to limit cycle behavior. Under this condition, explicit solvers have an advantage over implicit solvers in terms of computational cost because of the lesser number of operations required. In addition, LC behavior is dependent on the parameter set {s, q, w, f} and initial conditions for the species involved [9-12]. The choice of the parameter set and initial conditions is based on a previous solution [13] that has been verified to exhibit LC behavior. This implies that the parameter set used for this study may not be the best choice. Thus, the characterization of the parameter space for the Oregonator model remains a worthwhile problem.

The key advantage in selecting MATLAB numerical solvers for comparison is that they are widely used in engineering and science and these solvers' reliability is of critical interest to the community. For those experienced users who may implement these solvers individually, it is also critical for them to understand the intrinsic differences in all types of solves, so as to be able to tailor solver algorithms to specific problems. For instance, Cocherová modified the ode23 solver to solve the Hodge-Huxley model of electrical conduction through a nerve fiber [14]. Results from her work demonstrate improved accuracy and convergence of solutions even under RET.

The next step is to determine whether there exists an analytical system-model dependency, that is, to extract and map features of biological systems to concrete analytical models. This extraction process for biomolecular systems emulates the modeling process of conventional systems such as mechanical, electrical and fluid flow systems. The numerical solver selection results from this work should help in validating the analytical model and also in translating systems biology to clinical treatrment and therapeutic engineering applications.

## Conclusion

We have demonstrated that the selection of numerical solvers plays an important role in modeling outcomes. Using both qualitative and quantitative performance measures, we have shown that ode15 is an optimal implicit solver for solving stiff systems of ODEs, and enforcing strict relative error tolerance is a key factor for improving the performance of numerical solvers for convergent solutions. However, enforcing stricter relative error tolereance leads to an increase in computational costs. For explicit numerical solvers, ode45 is found to perform consistently regardless of relative error tolerance. These results reinforce the iterative process of biomolecular systems modeling, and indicate that it is not only possible to improve and ensure reproducibility of the analytical models, and also possible to improve the efficiency and effectiveness of experimental validations of these models.

## Methods

Here we discuss: (a) justification for the choice of the Belousov-Zhabotinskii (BZ) reaction as our test case, (b) the corresponding Oregonator model proposed by Field, Körös and Noyes, (c) stiff problems and (d) an overview of selected numerical solvers.

### Belousov-Zhabotinskii (BZ) reaction

The Belousov-Zhabotinskii (BZ) [HBrO_2_-Br--Ce(IV)] reaction [4,15,16] was chosen as the test system for three reasons:

1. The long-term objective of our research is to study biochemical systems modeling for human disease diagnosis and treatment. So, we want to choose a model that can be easily extended to clinical applications. Clinical disease conditions are often the consequence of cellular metabolite accumulations or deficiencies that arise from faulty cellular metabolite recycling mechanisms. Metabolites exhibit temporal oscillations in healthy cells as a result of consistent metabolite recycling. The BZ reaction model demonstrates similar sustained temporal oscillations i.e. limit cycle behavior that may be observed as a continuous progression of concentric waves in a bench-top flask. Furthermore, there is a correlation of the BZ reaction model and cell behavior. For instance, sustained temporal oscillations in a biochemical system are possible only if the system is maintained far from equilibrium i.e. the system is open and mass transfer occurs freely across the boundaries between the system and its surroundings. In cells, the cellular cytoplasm may be considered an open system to a limited extent because metabolic reactions occur in localized regions within the cytoplasm. Thus, the BZ reaction is a reasonable model to investigate that is readily extensible to clinical applications.

2. The BZ reaction is readily reproducible with materials found easily in classroom laboratories. As such, analytical or numerical predictions regarding the amplitude and frequency of oscillations can be easily verified. Furthermore, because the BZ reaction may be replicated in multiple settings [17] using different reactants with slightly different settings, there is a large variability, or a diverse family, of BZ reactions that are suitable and useful for validation of numerical solutions.

3. The BZ reaction is well-studied and modeled since the early 1970s when Field and Noyes first proposed the classic Oregonator [4,17] model to model the BZ reaction. As such, a wide array of literature [9-13] is readily available to provide an additional means to verify our results. While the Oregonator model may not be perfect, the model parameters and initial conditions are sufficiently well-characterized, making the Oregonator model an ideal choice for case study.

### Oregonator model [17]

From Field, Körös and Noyes, the Oregonator model is a set of 5 kinetic reactions with 2 substrates, 3 intermediates and 2 products as follows:

(1) *A *+ *Y *→ *X*

(2) *X *+*Y *→ *P*

(3) *B *+ *X *→ 2*X *+ *Z*

(4) 2*X *→ *Q*

(5) *Z *→ *fY*

where A, B are substrates; P, Q are products; X, Y and Z are intermediates; f is a stoichiometric factor.

The intermediates X, Y and Z, representing HBrO_2_, Br-, and Ce(IV) respectively, exhibit limit cycle behavior under suitable conditions. Assuming irreversible reactions (1–5), we express the rate of change of these intermediates as follows, in physical dimensions of concentration per time:

(6) $$\frac{dX}{dt}=k_{1}AY-k_{2}XY+k_{3}BX-2k_{4}X^{2}$$

(7) $$\frac{dY}{dt}=-k_{1}AY-k_{2}XY+k_{5}fZ$$

(8) $$\frac{dZ}{dt}=k_{3}BX-k_{5}Z$$

where the ki's represent reaction rate constants in equations (1–5).

Furthermore, Field and Noyes cast this system of nonlinear ODEs into a physically dimensionless system in terms of *α*, *η *and *ρ*. By removing physical dimensions from the systems of ODEs, the associated analytical difficulties in manipulating these physical dimensions of the Oregonator model are resolved as:

(9) $$\frac{d\alpha }{d\tau }=s\left(\eta -\eta \alpha +\alpha -q\alpha^{2}\right)$$

(10) $$\frac{d\eta }{d\tau }=s^{-1}\left(-\eta -\eta \alpha +f\rho \right)$$

(11) $$\frac{d\rho }{d\tau }=w\left(\alpha -\rho \right)$$

where

$$\begin{array}{ccc} X=\frac{k_{1}A}{k_{2}}\alpha & Y=\frac{k_{3}B}{k_{2}}\eta & Z=\frac{k_{1}k_{3}AB}{k_{2}k_{5}}\rho \\ s=\sqrt{\frac{k_{3}B}{k_{1}A}} & w=\frac{k_{5}}{\sqrt{k_{1}k_{3}AB}} & q=\frac{2k_{1}k_{4}A}{k_{2}k_{3}B} \end{array}$$

and f remains the stoichiometric factor (physically dimensionless). Thus, under a prescribed set of parameters {s, w, q, f}, the solutions to this physically dimensionless system, equations (9–11), derived from a variety of numerical solvers are of particular interest.

### Stiff problems

Stiff problems are problems where the speed of computing the numerical solution is restricted by the smallest step-size constrained by the parameter with smallest magnitude and dynamic range. As a result, it has significantly increased computational cost and potentially compromised the accuracy of the computed numerical solution. For example, in a previous study [13], a parameter set {s = 100, w = 3.835, q = 1 × 10^-6^, f = 1.1} and initial conditions {*α *= 20, *η *= 1.1, *ρ *= 20} were verified to lead the Oregonator model to exhibit limit cycle behavior. This set of parameters {s, w, q, f} differs significantly in orders of magnitude (i.e. q is in the order of 10^-6 ^in comparing to s in the order of 10^2^), which makes the system stiff where the solution step-size is constrained by the smallest parameter q.

To obtain a strong, unambiguous basis for comparison of numerical solvers, we use MATLAB to simulate this model with adaptive step-sizes for 200 time units using this a priori set of prescribed parameters and initial conditions.

### Numerical solvers

A variety of numerical solvers are available in MATLAB to deal with stiff, coupled, nonlinear systems of differential equations such as the physically dimensionless Oregonator model in terms of *α*, *η *and *ρ*. A brief overview of the available numerical solvers is summarized in Table 1[5,18-20].

From Table 1, there is a broad selection of numerical solvers, ranging from low to high order, single- and multi-step as well as explicit and implicit solvers implemented to deal with both stiff and non-stiff problems. The 3 explicit and 4 implicit numerical solvers selected for comparison represent key analytical and practical components of numerical solvers across the board.

A systematic study of the formulation and practical implementation constraints of these numerical solvers is conducted using specific performance measures such as convergence, accuracy and computational cost to summarize the efficacy of these numerical solvers. Evident from Table 1, there are multiple ways to characterize numerical solvers; here, the numerical solvers examined in this work are primarily organized and compared by the basis of ability to deal with stiff problems.

## List of abbreviations

BZ – Belousov-Zhabotinskii; LC – Limit cycle; RK – Runge-Kutta; RET – Relaxed relative error tolerance; SET – Strict relative error tolerance; ODE – Ordinary differential equation.

## Competing interests

The authors declare that they have no competing interests.

## Authors' contributions

CQ implemented the Oregonator model, gathered results and prepared the original draft. MW directs the whole project and publication.

## References

[B1] Hendriks BS, Opresko LK, S WH, Lauffenburger D (2003). Quantitative analysis of HER2-mediated effects on HER2 and epidermal growth factor receptor endocytosis – distribution of homo- and heterodimers depends on relative HER2 levels. J Biol Chem.

[B2] Larter R, Bush CL, Lonis TR, Aguda BD (1987). Multiple steady-states, complex oscillations, and the devil's staircase in the peroxidase-oxidase reaction. J Chem Phys.

[B3] Fitzhugh R (1960). Thresholds and plateaus in the Hodgkin-Huxley nerve equations. Journal of General Physiology.

[B4] Field RJ, Körös R, Noyes RM (1972). Oscillations in chemical systems. II. Thorough analysis of temporal oscillation in the bromate-cerium-malonic acid system. J Amer Chem Soc.

[B5] MATLAB ver 704 Help Documentation MATLAB.

[B6] Atkinson KE (1989). An Introduction to Numerical Analysis.

[B7] Faires JD, Burden R (2003). Numerical Methods.

[B8] Press WH, Flannery BP, Teukolsky SA, Vetterling WT (1990). Numerical Recipes in C.

[B9] Murray JD (1974). On a model for the temporal osciallations in the Belousov-Zhabotinskii reaction. J Chem Phys.

[B10] Tyson JJ (1977). Analytic representation of oscillations, excitability, and traveling waves in a realistic model of the Belousov-Zhabotinskii reaction. J Chem Phys.

[B11] Murase C, Sakanoue S (1983). Unstable and stable limit cycle in the Oregonator model for the Belousov-Zhabotinskii reaction. Prog Theor Phys.

[B12] Noyes RM (1984). An alternative to the stoichiometric factor in the Oregonator model. J Chem Phys.

[B13] Simulink implementation of the Oregonator model. http://www.mathworks.com/matlabcentral/.

[B14] Cocherová E (2003). Modification of ordinary differential equations MATLAB solver. Radioengineering.

[B15] Field RJ, Noyes RM (1972). Explanation of spatial band propagation in the Belousov reaction. Nature.

[B16] Belousov-Zhabotinskii Model. http://math.fullerton.edu/mathews/n2003/Belousov-ZhabotinskiiBiB.html.

[B17] Field RJ, Noyes RM (1974). Oscillations in chemical systems. IV. Limit cycle behavior in a model of a real chemical reaction. J Chem Phys.

[B18] Shampine LF, Reichelt MW (1984). The MATLAB ODE suite. SIAM Jour Sci Comput.

[B19] Shampine LF, Reichelt MW, Kierzanka JA (1999). Solving Index-I DAEs in MATLAB and SIMULINK. SIAM Review.

[B20] Hosea ME, Shampine LF (1996). Analysis and implementation of TR-BDF2. Appl Num Math.

